# A treatment revolution for those who can afford it? Hepatitis C treatment: new medications, profits and patients

**DOI:** 10.1186/1471-2334-14-S6-S5

**Published:** 2014-09-19

**Authors:** Maria Phelan, Catherine Cook

**Affiliations:** 1 Harm Reduction International, UK

## 

*“We are witnessing a revolution in the treatment of hepatitis C virus…. There is no question that these treatments that can save millions of lives must be made universally available at an affordable price.”* – Francois Barre-Sinoussi, President of the International AIDS Society [1]

Globally, approximately 184 million people are chronically infected with the Hepatitis C virus (HCV), the majority of whom reside in low- and middle-income countries [2]. HCV is a very common co-infection of HIV with an estimated four to five million people affected [3]. While HCV is a global epidemic it has a disproportionate effect on marginalised groups, in particular people who inject drugs (PWID). The global prevalence of HCV among PWID was estimated at 67% in 2010, with more than 10 million PWID living with the virus [4]. Moreover, among PWID who are living with HIV, approximately 75% are co-infected with HCV [5]. While the burden of HCV falls disproportionately upon people who inject drugs, treatment coverage among this group remains extremely low, estimated at just 2%-4% of those eligible for treatment [6]. This is similar to the 4% coverage of antiretroviral therapy among PWID who are living with HIV [7].

## A treatment revolution?

While both HIV and Hepatitis C are bloodborne viruses, Hepatitis C is curable. Until recently, the standard of care was pegylated interferon (PEG-IFN) and ribavirin (RBV), but this regimen has significant drawbacks, including the requirement of a long treatment duration (24 to 48 weeks or longer) and frequent and sometimes severe side effects from both drugs [8]. Moreover, cure rates are low, at around 50% in low- and middle-income countries [9]. Recent developments in direct-acting antivirals (DAAs) to treat HCV have been hailed as revolutionary [10], and adding a first-generation DAA to PEG-IFN/RBV increased cure rates in genotype 1 patients to 75% [11]. (Genotype 1 is the most prevalent HCV genotype globally, and traditionally the most difficult to cure.) Newer-generation DAAs have brought cure rates up to 90% or higher and are easier to take [12]. Within the next couple of years, it is expected that all-oral DAA combinations with few side effects, taken for 8 to 12 weeks, will cure most people with HCV including those considered “hard to treat [13].” Many pharmaceutical companies are producing HCV DAAs and there are dozens of additional treatment candidates in the drug development pipeline. Several products, including Gilead’s sofosbuvir and Janssen’s simeprevir, have recently been approved in the United States and Europe [14].

## Profits before patients?

A substantial body of literature suggests that HCV is treatable among PWID and that, with the right support, adherence levels among PWID are equal to those of their non-drug-using peers [15]. Many of the barriers that exclude this group from treatment are structural or are based on stigma and discrimination [6]. However another significant barrier for all people with HCV, particularly in relation to new medications, is cost. Eighty-five percent of Hepatitis C patients reside in low- and middle-income countries, many of which have meagre health budgets, and patients in these countries generally are expected to pay for medications out-of-pocket [16]. Pegylated interferon, at around US$ 20,000 per course of treatment, has proven to be too expensive for people in many countries [16]. Sofosbuvir, which is expected to be the mainstay of HCV therapy for the near future, has the potential to benefit many more patients when combined with other medications, but its cost of US$ 84,000 per 12-week course (with comparable prices in Europe) will keep it out of reach for the majority of hepatitis C patients for the foreseeable future [16,17].

Given that the market for sofosbuvir will have an estimated worth of around US$ 9 billion by 2017 [18], civil society organisations and treatment advocates have called for a reduction in the price of new medications to US$ 500 per course [19].

Some progress has already been made through advocacy: in Egypt, which has the highest HCV prevalence in the world at 14% in the general population [20], the production of generic medications to rival brand-name competitors has allowed the government to secure prices of just US$ 2,000 per course of PEG-IFN [16]. Most recently the government negotiated a 99% discount on the US price of sofosbuvir, bringing it down to just US $900 per 12-week course [21].

Moreover, India’s Natco Pharma Limited has requested that the Indian patent office deny Gilead a patent for sofosbuvir in India, arguing that the new medication is not “innovative enough” and relies upon “old science.” If this request is approved, it will pave the way for the generic production of the drug [22]. Most recently, the World Health Organization issued a strong statement within new guidelines for hepatitis C treatment recommending the use of new medications, including sofosbuvir, and urging a reduction in price [23].

## What does this mean for Europe?

While the introduction of these new drugs constitutes one of the most important medical breakthroughs in recent years, the exorbitant cost means that even within Europe they are unlikely to be made available to PWID. In most Eurozone countries, sofosbuvir will be priced at EUR 50,000 to 60,000 per 12-week course [24].

A treatment gap is already evident for less costly hepatitis C treatment within the European Union: large variations have been reported between the economically stable countries in northern and western Europe and those greatly affected by the 2009 financial crisis in southern and eastern Europe. It was estimated that 6.7% of hepatitis C patients in France undertook treatment in 2010, compared to just 0.8% in Italy [25]. The treatment rate was also very low in Poland (0.4%), Romania (1.0%) and Russia (0.3%), and it has been suggested that the cost of treatment was a factor [25].

Even in high-income countries, the cost of new treatment is likely to have an impact on uptake. For example, in France it has been estimated that the price charged by Gilead is 756 times the cost of production of sofosbuvir [17]. With 127,500 chronic HCV patients [17] within France who could benefit from sofosbuvir, the high cost virtually guarantees that this is not a sustainable treatment option.

Twenty-one countries within Central Asia and Eastern Europe are currently classified as having middle-income status [26]. These countries, which are excluded from licensing agreements with Gilead, have small health budgets and will be unable to purchase these treatments [24].

Due to the withdrawal of the Global Fund to Fight AIDS, Tuberculosis and Malaria and other international donors, harm reduction programmes are under threat in countries such as Bulgaria, Georgia and Moldova [27]. Not only did Global Fund support ensure the continuation of harm reduction services in the past, but it also was a factor in the negotiation of lower hepatitis C medication prices in countries such as Ukraine [28].

## Recommendations

Gilead should drastically reduce the price of sofosbuvir. Doing so will promote access to this lifesaving treatment. It has also been suggested that sales would increase and profits would stabilise [24]. Gilead should also commit to an early access programme for European countries whose healthcare systems are unable to afford sofosbuvir [24].

Generic competition must be fostered; the recent developments in India are paving the way for this dynamic to emerge. The success seen in price reductions for HIV medications as a result of the introduction of generic products should provide a model upon which HCV treatment advocates can expand.

Within those countries in the European region that are still eligible for Global Fund support, the Global Fund should increase its attention to hepatitis C. While the Global Fund does not have a specific strategy for increasing access to hepatitis C prevention, treatment and care, it does have supportive guidance. In countries with “well-documented evidence that hepatitis C treatment and funding is available to the general population,” Global Fund monies can be used to “fill in the gap for HIV-infected individuals [29].” The Technical Review Panel has recommended that Global Fund resources be used to amass evidence on the need for hepatitis treatment, create awareness of the virus, increase prevention efforts, and support advocacy for treatment access and affordability [30]. The Global Fund could draw on the experience accumulated in programmes focusing on HIV/HCV co-infection, such as in Georgia and Ukraine, where negotiations regarding the cost of medications have led to substantial price reductions.

European civil society should learn from how reductions in the prices of HIV medications have been achieved and should apply similar pressure in relation to hepatitis C medications. A coordinated effort from a variety of stakeholders in Europe should be undertaken. Promising developments such as those seen mostly recently in Egypt and India around generic production should be encouraged and extended.

Civil society should be closely involved in HCV policy and programme development. In particular, the meaningful engagement of people who use drugs is key. Funding should also be directed towards these groups as a priority.

Innovative advocacy and coordinated efforts by civil society have resulted the formation of an HCV civil society reference group at the World Health Organization and the inclusion of PEG-IFN on the list of essential medicines [31]. These strategies should be leveraged within Europe to ensure that PWID there can also access new and emerging treatments.

## Competing interests

The authors declare that they have no competing interests.

## Declarations

This article has been published as part of *BMC Infectious Diseases* Volume 14 Supplement 6, 2014: Viral Hepatitis in Europe. The full contents of the supplement are available online at http://www.biomedcentral.com/bmcinfectdis/supplements/14/S6. The publication charges for this supplement were funded by AbbVie as an unrestricted grant to Rigshospitalet, the University of Copenhagen. AbbVie further funded the printing of the supplement with additional financial support from the Drug Prevention and Information Programme (DPIP) of the European Union.
